# How Bacteria Stopped Worrying and Learned to Love…Formaldehyde

**DOI:** 10.1371/journal.pbio.0030055

**Published:** 2005-01-04

Poring over the vast amount of sequence and genetic information now available for many organisms, scientists frequently encounter what appear to be redundant biochemical pathways. Redundant pathways take the same starting material and transform it into the same product, but through different routes. Why should cells maintain redundant pathways?

An interesting case is that of Methylobacterium extorquens, a bacterium that can grow on organic molecules with a single carbon atom such as the alcohol methanol. Bacteria in this species first oxidize methanol into formaldehyde, then use formaldehyde to make serine—the entry point for the synthesis of many of the cell's building blocks—via two apparently redundant pathways. The short pathway is a direct (non-enzymatic) reaction of formaldehyde with tetrahydrofolate to make methylene-tetrahydrofolate, which donates a single carbon atom for serine synthesis. A hypothesized long pathway could also lead to methylene-tetrahydrofolate through a long series of enzymatic reactions, one of which consumes energy.

Christopher Marx and colleagues now demonstrate that the bacteria modulate their use of each pathway during the course of acclimation to growth on methanol. In the process the authors offer new insights into the bacteria's rapid disposal of formaldehyde, a toxic chemical that would pickle cells in a minute if it was allowed to accumulate.[Fig pbio-0030055-g001]

**Figure pbio-0030055-g001:**
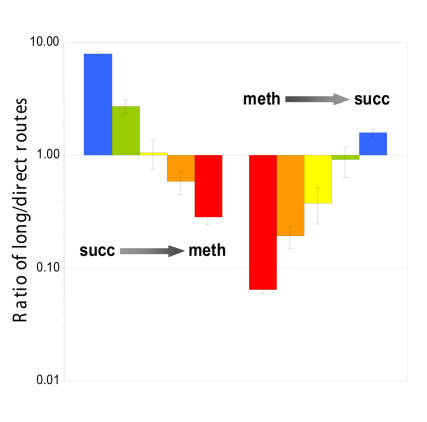
The short and long (pathways) of formaldehyde metabolism

The authors noticed that the short pathway transferred both hydrogen atoms of formaldehyde to the serine molecule while the long pathway transferred only one. If they fed bacteria methanol in which hydrogen had been replaced with its heavier form, deuterium: the resulting serine was slightly heavier than normal, as a result of acquiring one—or two—deuterium atoms.

By measuring the ratio of the two serine forms, Marx and colleagues inferred the relative contribution of the two pathways to serine synthesis. The long pathway dominated, accounting for eight times more serine than the short pathway, when cells first encountered methanol. But the situation was reversed after the cells acclimated to methanol: then the short pathway produced 15 times more serine than the long pathway.

The authors also measured absolute amounts of formaldehyde processed by each pathway, using methanol marked with a heavy form of carbon (^14^C). Although the relative contribution of the long pathway decreased during ramping-up to methanol growth, the absolute amount of formaldehyde that flowed through it increased 8-fold within the first half of the transition, and then decreased.

The authors generated a mathematical model based on known reaction rates from the short and long pathways. When they simulated methanol exposure, the model predicted a switch from long to short pathway very similar to what they had observed experimentally.

The authors conclude that the pathways are not in fact redundant, but fulfill different functions. The long pathway is not an efficient means of serine synthesis from formaldehyde; in fact, the small amount of serine it produces is at some energy cost. But it allows the cell to spend ATP—the molecular fuel—to jump-start formaldehyde assimilation while avoiding formaldehyde accumulation when the cells first experience methanol. The short pathway is a direct and efficient (energy-free) route to serine production (and hence growth), but one that is slower to reach its cruising speed. Thus, the cells use these two formaldehyde assimilation routes like a driver uses the transmission of a car: start with powerful low gears when first accelerating, and shift to more efficient gears once hurtling down the road.

The combination of both pathways represents an elegant solution to the problem of growth in toxic environments and provides a useful paradigm for detoxification in medical and environmental contexts.

